# Upregulated but insufficient generation of activated protein C is associated with development of multiorgan failure in severe acute pancreatitis

**DOI:** 10.1186/cc3966

**Published:** 2006-01-13

**Authors:** Outi Lindstrom, Leena Kylanpaa, Panu Mentula, Pauli Puolakkainen, Esko Kemppainen, Reijo Haapiainen, Jose A Fernandez, John H Griffin, Heikki Repo, Jari Petaja

**Affiliations:** 1 Second Department of Surgery, Helsinki University Central Hospital and University of Helsinki, Finland; 2 Department of Molecular and Experimental Medicine, The Scripps Research Institute, La Jolla, CA, USA; 3 Department of Medicine, University of Helsinki, Finland; 4 Department to Bacteriology and Immunology, Haartman Institute, University of Helsinki, Finland; 5 Department of Pediatrics, Jorvi Hospital, Espoo, Finland

## Abstract

**Introduction:**

Disturbed protein C (PC) pathway homeostasis might contribute to the development of multiple organ failure (MOF) in acute pancreatitis (AP). We therefore evaluated circulating levels of PC and activated protein C (APC), evaluated monocyte deactivation in AP patients, and determined the relationship of these parameters to MOF.

**Patients and methods:**

Thirty-one patients in the intensive care unit were categorized as cases (*n *= 13, severe AP with MOF) or controls (*n *= 18, severe AP without MOF). Blood samples were drawn every second day to determine the platelet count, the levels of APC, PC, and D-dimer, and the monocyte HLA-DR expression using flow cytometry. The APC/PC ratio was used to evaluate turnover of PC to APC.

**Results:**

During the initial two weeks of hospitalization, low PC levels (<70% of the adult mean) occurred in 92% of cases and 44% of controls (*P *= 0.008). The minimum APC level was lower in cases than in controls (median 85% versus 97%, *P *= 0.009). Using 87% as the cut-off value, 8/13 (62%) cases and 3/18 (17%) controls showed reduced APC levels (*P *= 0.021). A total of 92% of cases and 50% of controls had APC/PC ratios exceeding the upper normal limit (*P *= 0.013). Plasma samples drawn before MOF showed low PC levels and high APC/PC ratios. HLA-DR-positive monocytes correlated with PC levels (*r *= 0.38, *P *< 0.001) and APC levels (*r *= 0.27, *P *< 0.001), indicating that the PC pathway was associated with systemic inflammation-triggered immune suppression.

**Conclusion:**

PC deficiency and decreased APC generation in severe AP probably contributed to a compromised anticoagulant and anti-inflammatory defence. The PC pathway defects were associated with the development of MOF. The data support feasibility of testing the use of APC or PC to improve the clinical outcome in AP.

## Introduction

Acute pancreatitis (AP) is a common disease with widely variable clinical outcome. Twenty-five per cent of patients suffer from the severe form of the disease with local and/or systemic complications, resulting in a mortality rate ranging from 2 to 10% [1]. Increased morbidity and mortality are associated with organ failure in 50% of severe AP cases [2].

Systemic inflammatory reaction and the development of organ failure in AP share similarities with complicated courses of sepsis, major trauma, and burns [3]. In systemic inflammation, excessive proinflammatory burst is rapidly followed by an anti-inflammatory reaction that may result in immune suppression [4]. Likewise, rapid activation of coagulation may turn into global or selected exhaustion of physiological anticoagulant systems. In sepsis, for example, coagulation, inflammation, and apoptosis all contribute to organ dysfunction and permanent damage. The interactions between coagulation and inflammatory pathways are essential in the pathogenesis of disseminated intravascular coagulation. For example, the proinflammatory cytokines tumour necrosis factor alpha, IL-1, and IL-6 upregulate thrombin formation and downregulate physiological antithrombotic defence mechanisms, especially the protein C (PC) pathway [5].

The PC pathway is both a major physiological anticoagulant system and a central link between inflammation and coagulation. The zymogen PC is converted to an active serine protease activated protein C (APC) by thrombin bound to thrombomodulin on the endothelial surface [6]. This effect is enhanced by the endothelial PC receptor [7]. APC conveys its anticoagulant function mainly by proteolytic inactivation of coagulation activated factor V and activated factor VIII. APC also exhibits distinct anti-inflammatory and anti-apoptotic properties [8-11]. While the underlying mechanism remains incompletely understood, recombinant APC decreased the levels of IL-6 and D-dimer and reduced mortality in severe sepsis patients [12].

Few studies have explored systematically haemostatic disturbances during AP [13-15]. An increase in plasma-soluble thrombomodulin predicted a lethal course of AP [16]. No data on APC in AP patients have been published. Based on the central role of the PC pathway in the acute systemic inflammatory response and the availability of two therapeutic approaches, zymogen PC concentrate [17] and recombinant APC [12], we decided to evaluate how the PC pathway evolves during the course of severe AP. In the current study we tested the hypothesis that a failure of the PC pathway homeostasis might be involved in the development of organ failure in AP patients.

## Patients and methods

### Patients

The study population consists of 31 patients with AP treated in the intensive care unit at Helsinki University Central Hospital between April 2001 and February 2003. The study protocol was approved by the local ethics committee and informed consent was obtained from each patient. Diagnosis of AP was based on elevated serum amylase concentration (at least twofold higher than the upper reference limit) and/or a typical appearance of AP on computed tomography associated with typical clinical findings, including acute abdominal pain. The patients had severe AP according to the Atlanta classification [18], in which severe AP is associated with organ failure and/or local complications such as necrosis, abscess, or pseudocyst. Computed tomography was performed on all patients.

Organ failure was defined as the development of respiratory failure necessitating mechanical ventilation and/or renal failure necessitating haemodialysis. The criteria for initiating mechanical ventilation were tachypnea (respiratory rate >35/min) and/or the need for an inspiratory oxygen fraction >0.6 in order to maintain an arterial partial pressure of oxygen >8 kPa. Haemodialysis was started in patients with significantly impaired renal function as indicated by increased concentrations of serum creatinine (>300 μmol/l) and serum urea (>40 mmol/l) and progressive metabolic acidosis (pH < 7.28) in serial measurements regardless of urine output.

Organ failure developed in 13 patients (case group). The remaining 18 patients, all of whom met the criteria of severe AP but did not develop vital organ dysfunction, served as controls (control group). The characteristics of the two patient groups are presented in Table 1. Values for routine coagulation parameters (prothrombin time [PT], D-dimer, and platelet count) were recorded from the hospital records. Blood samples were taken for the study purpose every second day during the patient's stay in the intensive care unit.

### Protein C and activated protein C

Plasma levels of PC and APC were determined by enzyme capture assay, as previously described [19]. Briefly, a monoclonal antibody against PC and APC was immobilized in microplates. Plasma samples supplemented with benzamidine, a reversible inhibitor of thrombin, APC, and trypsin-like proteases, were incubated in the wells for the capture of APC and PC. The plates were then washed to remove sample constituents and benzamidine. The amidolytic activity of the captured APC was measured with chromogenic substrate S-2366 (Chromogenix AB, Mölndal, Sweden). Assays were run in duplicate, and a noncommercial plasma pool containing benzamidine was used as the standard. The sensitivity of the assay is five pmol/l, corresponding to 13% of the normal mean plasma level of APC in healthy resting adults [19].

The total PC was measured by activating the bound PC in the wells by Protac (American Diagnostica, Greenwich, CT, USA) and then measuring the amidolytic activity with the chromogenic substrate S-2366 (Chromogenix AB). The amidolytic activity observed after Protac activation is essentially equal to the total PC. Assays were run in duplicate. The results of APC and total PC are expressed as a percentage relative to the plasma pool from healthy adults, defined as 100%. The reference range (± 2 standard deviations) for APC is 44–200% and that for APC/PC is 0.64–1.48 [20]. For PC, 70% of the normal adult mean was used as the lower limit of normal.

### Flow cytometry

APC acts *in vitro *as an anti-inflammatory agent largely through modulating monocyte activation during inflammation [9-11]. Monocyte HLA-DR expression reflects the recent history of activation/functional suppression of the circulating monocyte population [21]. HLA-DR was determined using whole blood flow cytometry, as described previously [4,21]. The mAbs were phycoerythrin-conjugated anti-HLA-DR mAb (IgG2a, clone L243), phycoerythrin-conjugated irrelevant mAb (IgG2a, clone X39), and fluorescein isothiocyanate-conjugated anti-CD14 mAb (IgG2b, clone MFP9) (Becton Dickinson, San Jose, CA, USA). HLA-DR expression is defined as the proportion of positively fluorescing monocytes (HLA-DR%).

### Statistics

All data are expressed as medians and ranges. Comparisons of marker levels between the two groups were performed by the Mann-Whitney *U* test. Fisher's exact test or chi-square tests were used to compare the proportions of patients between the two groups when appropriate. Spearman's rank correlation was used for assessing correlations. Receiver operating characteristics analysis was used for determination of an optimal cut-off level for APC in differentiating cases from controls. Comparisons of the follow-up samples were performed with Friedman's test, followed by Dunn's test for post-hoc comparisons when appropriate. SPSS 12.0.1 for Windows (ACITS; The University of Texas at Austin, Austin, TX, USA) statistical software was used. *P *< 0.05 was considered statistically significant.

## Results

### PC pathway during hospital care

**Figure 1:**
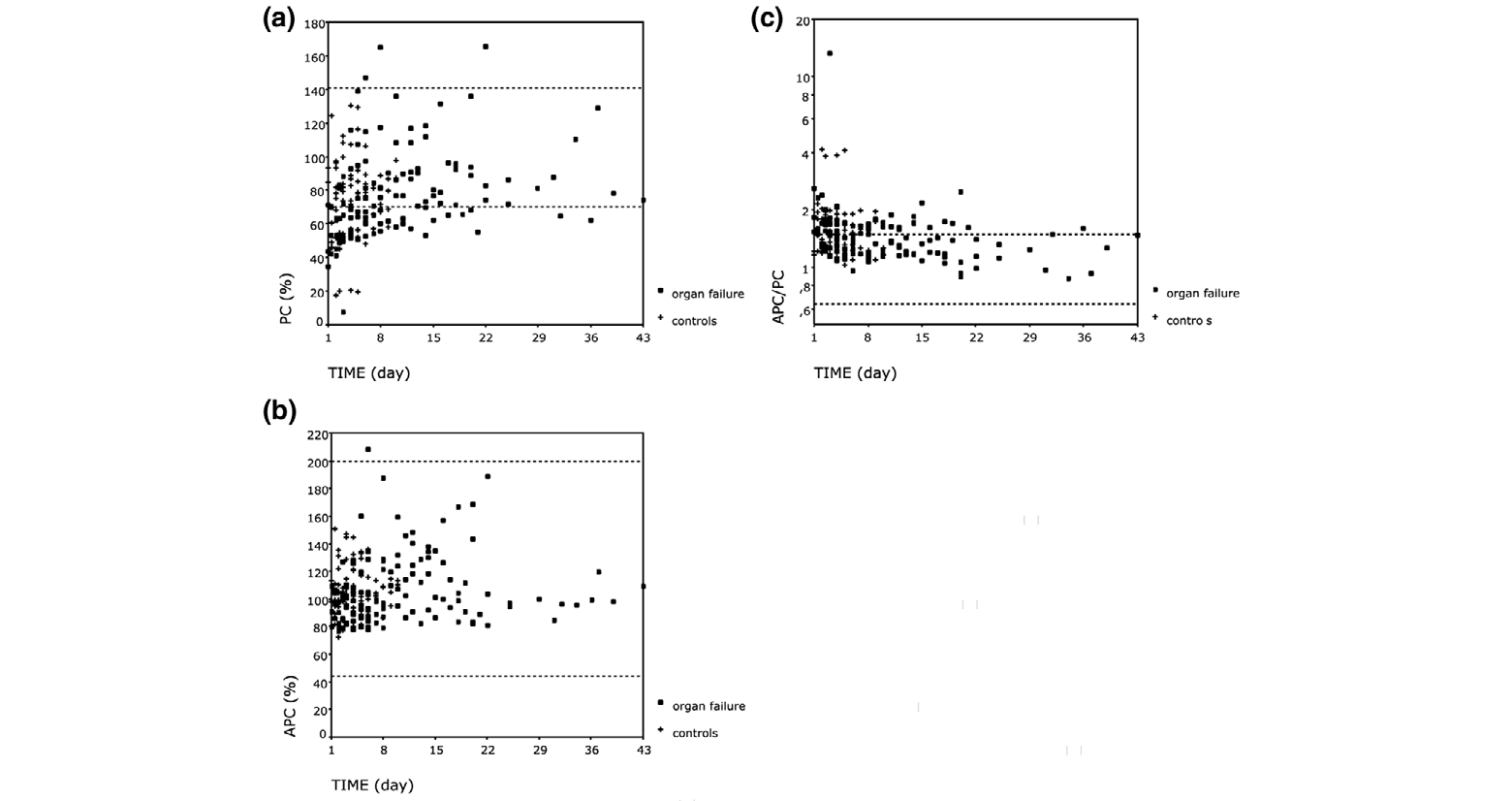
Levels of **(a) **protein C (PC), **(b) **activated protein C (APC), and **(c) **APC/PC ratio during hospital stay in patients with acute pancreatitis. Control, patients with acute pancreatitis without multiple organ failure. The dotted lines indicate reference values for healthy individuals. Day 1 indicates the day of admission to the research hospital

During the observation period all patients showed evidence for activated haemostatic system. The levels of D-dimer were elevated by 2.2-fold to 95-fold compared with the normal upper limit of 0.5 mg/l. All values of PC levels, APC levels, and the APC/PC ratio during the hospital stay are shown in Figure 1a–c.

Decreased PC values (defined as <70% of the normal plasma pool) were a frequent finding; found in 43% of all samples (68% of all patients) at various stages of the disease (Figure 1a). The intrapatient variation in PC levels from day to day was large. However, no association between the time of sampling and the PC levels was found. Administration of blood products did not readily explain the intrapatient variation of PC levels (data not shown).

The levels of APC showed much less variation than did the PC levels. All but one of the APC values fell within the range observed in healthy resting adults (Figure 1b). As for PC, no general dependency of APC levels with respect to the timing of the samples was observed.

The rate of conversion of PC to APC was estimated by calculating the APC/PC ratios, for which we previously defined a preliminary normal range [20] (Figure 1c). The APC/PC ratio did not fall below the lower limit of normal in any sample but exceeded the upper normal limit in 40% of the samples. At least one APC/PC ratio value was above normal in 74% of all patients.

Associations between general coagulation screening parameters and PC pathway components were analysed. APC levels correlated positively with the PT (*r *= 0.28, *P *= 0.01) and with the platelet count (*r *= 0.32, *P *= 0.01), as did the PC concentration (*r *= 0.48 for PT and *r *= 0.32 for platelet count, respectively; both *P *= 0.01). The APC/PC ratio correlated negatively with the PT (*r *= -0.43, *P *= 0.01). The D-dimer levels did not correlate with APC levels (*r *= 0.15, *P *= 0.08), PC levels (*r *= 0.14, *P *= 0.10), or the APC/PC ratio (*r *= -0.06, *P *= 0.48).

### Cases versus controls

#### Samples on admission

Samples on admission (defined as sampling within 36 hours of actual admission) were available from 11 cases and 15 control patients. The APC concentration was significantly lower in cases than in controls (median APC 86% versus 105%, *P *= 0.027), whereas the PC level and the APC/PC ratio did not differ significantly between the two groups. However, 89% of cases and 43% of controls showed an abnormally high APC/PC ratio (*P *= 0.04).

#### Follow-up samples

**Figure 2:**
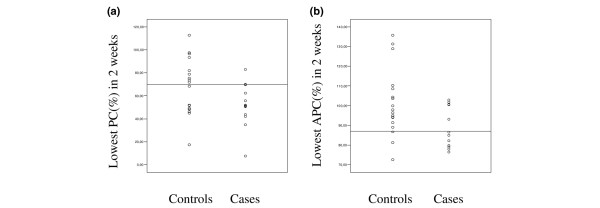
The lowest **(a) **protein C (PC) and **(b) **activated protein PC (APC) values during the first two weeks of hospital care in acute pancreatitis patients with multiple organ failure (cases) and in those without organ failure (controls). *P *= 0.055 and *P *= 0.022 for PC and APC, respectively; Mann-Whitney *U* test.

Multiple organ failure (MOF) developed from -2 to 14 days (median, 1 day) after admission to the research hospital. We compared the levels of the two PC pathway components between cases and controls during this time period (Figure 2). A decreased PC level was observed in 92% of cases and in 44% of controls (*P *= 0.008). The minimum (for instance the lowest observed value) PC level was lower in cases than in controls but the difference was not statistically significant (*P *= 0.055). The minimum observed APC level was lower in cases than in controls (median 51% versus 73%, *P *= 0.022; Figure 2b). Utilizing receiver operating characteristics analysis, the optimal cut-off value for APC to differentiate cases from controls was found to be 87%. Eight out of 13 (62%) patients with MOF showed a minimum APC level below this limit, while the same was true for 3/18 (17%) of controls (*P *= 0.021). During the first 2 weeks of hospitalization, 92% of cases and 50% of controls had APC/PC ratios exceeding the upper normal limit (*P *= 0.020).

The results of coagulation parameters during the stay in the intensive care unit are presented in Table 2. There were no differences in D-dimer level, the PT, or the platelet count between cases and controls during the hospitalization. When only the samples from the first 14 days were analysed, however, the maximum D-dimer level was higher (median 11.7 mg/l versus 6.3 mg/l, *P *= 0.01) and minimum platelet count was lower (105 × 10^9^/l versus 171 × 10^9^/l, *P *= 0.001) in cases than in controls, respectively. The minimum PT was equal in the two groups during the first 14 days.

Because MOF was diagnosed at various time points relative to hospital admission, samples preceding the diagnosis of organ failure were available from only five patients. The PC level was below 70%, the APC level was within the normal range, and the APC/PC ratio was above normal in all these patients. When the pre-MOF samples of these five patients were compared with control samples, the median APC value was equal (*P *= 0.56), the median PC value was lower (*P *= 0.025), and the APC/PC ratio was higher (*P *= 0.02).

**Figure 3:**
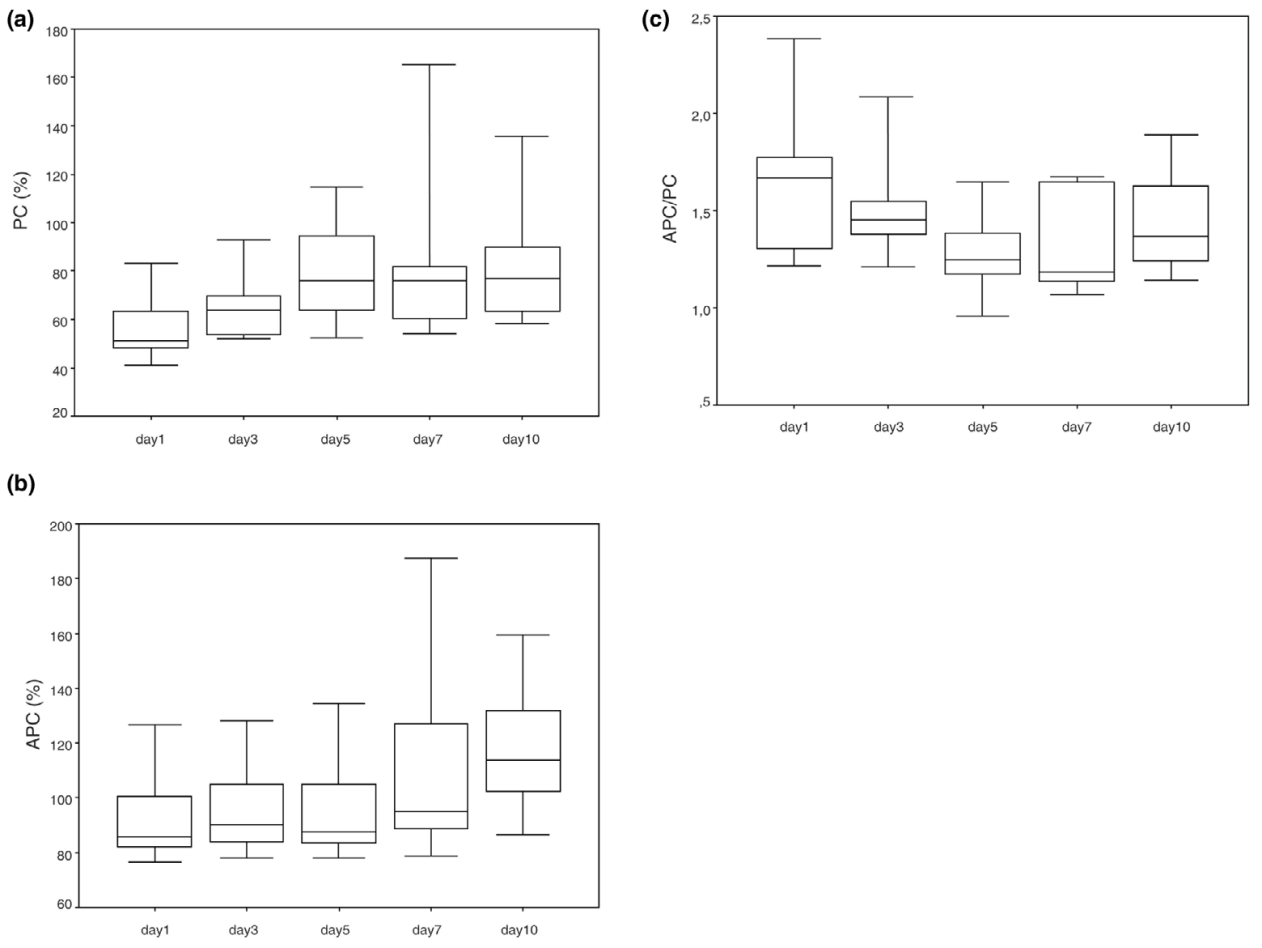
Follow-up concentrations of **(a) **protein C (PC), **(b) **activated protein C (APC), and **(c) **APC/PC ratio during the stay in hospital in nine patients with organ failure. There was no significant influence of time of sampling on any parameter (Friedman's test *P *> 0.05).

We also evaluated whether the time spent in hospital care could affect the measured parameters in the subgroup of patients with MOF. For this purpose, only patients with a sufficient number of follow-up samples were analysed. Follow-up samples with a maximum 2-day interval for 10 days were available from nine cases. During this 10-day period both the APC and the PC levels tended to increase (Figure 3a,b). The median APC/PC ratio was the lowest on day 7. The apparent mechanism for the decreasing APC/PC ratio during hospitalization was a gradual improvement of PC levels without a concomitant increase in APC values (Figure 3a–c). There was thus a trend of gradual improvement of early PC pathway disturbances during the course of MOF.

There were three deaths among the 13 patients in the case group. In these three nonsurvivors the median level of APC was 105% (85–188), that of PC was 74% (8–165), and that of the APC/PC ratio was 1.45 (1.13–13.2). In the 10 survivors of the case group, the median level of APC was 98% (76–109), that of PC was 71% (35–165), and that of the APC/PC ratio was 1.32 (0.87–2.6).

### HLA-DR% and the protein C pathway

**Figure 4:**
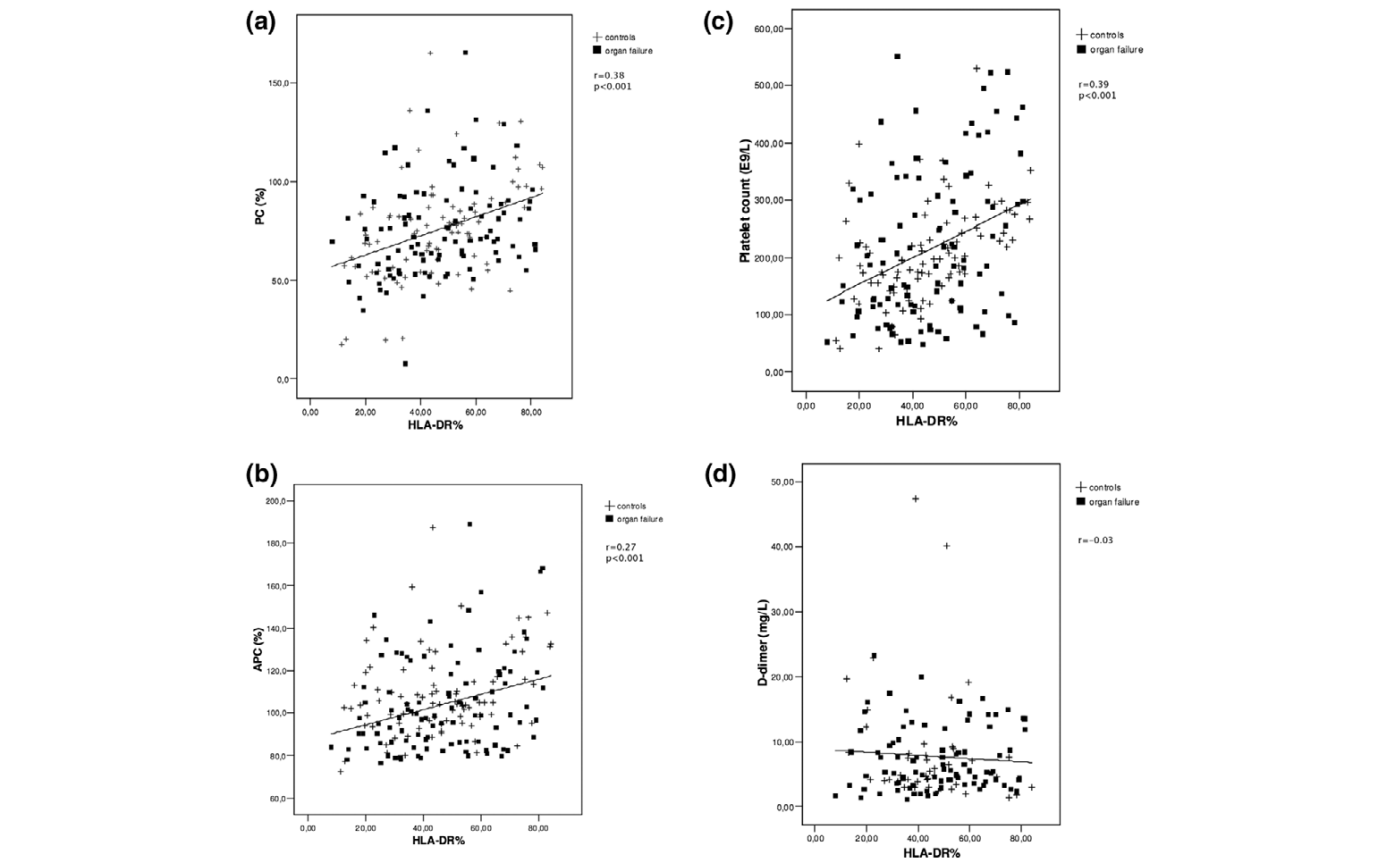
Correlations between percentage of HLA-DR-positive monocytes and **(a) **protein C (PC), **(b) **activated PC (APC), **(c) **platelet count, and **(d) **D-dimer concentrations. Correlation coefficients are presented for combined data of organ failure and control patients. E9, × 10^9^.

There was no difference in HLA-DR% between cases and controls during their stay in the intensive care unit (Table 2). During the first 14 days in the intensive care unit, however, the lowest HLA-DR% tended to be lower in cases (26%) than in controls (35%) (*P *= 0.095). Positive correlations between HLA-DR% and the PC concentration (*r *= 0.38, *P *< 0.001), the APC concentration (*r *= 0.27, *P *< 0.001), and the platelet count (*r *= 0.39, *P *< 0.001) were observed. HLA-DR% did not correlate with the PT (*r *= 0.12) or the D-dimer level (*r *= -0.03) (Figure 4a–d).

## Discussion

The current observational study of the course of PC and APC levels during AP in 31 patients with a 42% incidence of MOF was conducted to address how often and to what extent the PC pathway would be disturbed and whether such perturbations would be associated with the development of MOF. The study setting inevitably resulted in limitations in interpreting the results in terms of causality. For example, the patients entered the university hospital at various stages of AP, some having MOF developed prior to the admission. Also, despite our continuous efforts during the two years of patient recruitment, the sampling schedule in the original protocol was not completely fulfilled. The patient series was rather uniform in disease severity and the incidence of MOF approached 50%, however, giving us the opportunity to test the hypothesis of whether a defective PC pathway would be detrimental to patients with AP.

The minimum levels of APC in MOF patients were significantly lower than in controls. The PC deficiency was therefore logically associated with a decrease of APC levels in patients with MOF. A more significant finding, however, may be that the APC levels seemed not to be grossly elevated. In fact, only one sample in one patient showed an absolute APC level that exceeded the reported upper limit of normality (200%) [20]. Thrombin is the APC activator, and APC is a feedback inhibitor for thrombin generation [22,23]. The complicated balance between thrombin, PC, and APC varies depending on the circumstances. In resting healthy adults, the PC level rather than the thrombin level may be the major determinant of the circulating APC level [20,24,25]. During cardiopulmonary bypass, upon rapid thrombin generation during reperfusion, the APC/PC correlation was lost and a significant positive correlation between fibrinopetide A, a thrombin marker, and APC developed [24].

The upper limit for APC formation may be estimated from APC determinations in various clinical settings where coagulation is known to be activated. In our previous studies the most pronounced and fast enhancements from normal resting APC levels to levels typically ranging from 250% to over 800% of the normal mean were observed during the first minutes of reperfusion in liver transplantation [26] and during an infusion of antithymocyte globulin, a strong proinflammatory stimulus, in renal transplantation [27]. Liaw and colleagues recently reported that in acute sepsis patients, despite ongoing activation of coagulation, 25% of patients failed to increase their APC levels above 250% (while 75% had levels ranging from approximately 250 to 800%) [28]. They concluded that the septic patients varied markedly in their ability to generate APC in response to the physiological thrombin stimulus, and attributed the phenomenon to endothelial dysfunction [28]. In children with meningococceal sepsis, on the other hand, patients' ability to generate APC in response to thrombin did not seem to be grossly affected as infusion of PC resulted in elevated levels of APC, and significant correlations were observed between APC and thrombin markers at baseline [17].

Even though D-dimer is not a direct thrombin marker, the highly elevated D-dimer levels in the current study indicated that ample thrombin formation occurred throughout the observation period. Therefore, in good accordance with the study by Liaw and colleagues in adult septic patients [28], we assume that the lack of elevated free APC levels in the presence of activated coagulation in patients with AP in most cases reflected dysfunctional PC activation on the endothelium, or possibly enhanced inhibition of APC by plasma protease inhibitors. This suggestion, however, does not exclude the possibility that significant PC deficiency could also be rate limiting for APC formation in patients with severe AP, as seems to be the case in septic children [17].

The PC levels were subnormal in 92% of patients with MOF and in 44% of controls. Low PC was found to precede MOF, and early APC/PC ratios were frequently high in MOF patients. In an animal model of AP, the PC level was found to decline already one hour after initiation of AP [29]. The PC levels often fall rapidly in sepsis patients [30,31], and this may associate with the development of organ failure [32]. Thus, in logical accordance with animal AP data and human sepsis studies, early PC deficiency was a most frequent finding during severe AP. While the causal relationship between low PC levels and MOF development cannot be proven in an observational study setting, one probable bias could be excluded. Namely, there was no indication that PC deficiency would associate with MOF after its diagnosis and, further, there was a significant trend for improvement of PC homeostasis during the course of MOF. It thus seems that, in patients developing MOF, the PC activation system reached its limits prior to the diagnosis of MOF, resulting in secondary deficiency of PC concomitantly with a failure to maintain steady APC levels similar to those observed in AP patients without MOF.

APC and PC correlated significantly with monocyte HLA-DR expression but failed to associate with the level of D-dimer. The existence of anti-inflammatory and anti-apoptotic properties of APC is currently widely accepted [22,33]. Compared with the accumulating *in vitro *and animal data, however, observational human clinical studies provide scarce data on the specific relationship between APC and inflammation. The obvious limitation is the fact that thrombin formation is also enhanced whenever APC generation is enhanced, and the question remains whether APC or other components of the activated coagulation pathways might be involved.

The concomitant decrease in D-dimer and IL-6 during APC infusion in the PROWESS trial has been taken as circumstantial evidence of specific anti-inflammatory action of APC [12]. The remaining human studies finding associations between APC and inflammatory phenomena have involved only hyperacute proinflammatory/procoagulant situations such as reperfusion after cardiopulmonary bypass [34], liver transplantation [26], and renal transplantation [27]. We chose to measure HLA-DR%, which reflects the recent history of activation/functional suppression of the circulating monocyte population [21]. APC acts as an anti-inflammatory agent *in vitro*, largely through modulating monocyte activation during inflammation [9-11]. The current correlations between HLA-DR expression and the PC and APC levels may therefore support the concept of interaction between inflammation and the PC pathway. In severe AP, levels of PC and APC were moderately associated with monocyte activation status.

The current study suggests that testing the therapeutic use of APC or PC to improve patient outcome might be feasible in AP. The concurrent activation of inflammation and coagulation during AP, the frequent occurrence of PC pathway defects in AP patients, their association with a significant clinical endpoint (MOF), and the timing of major PC defects to the early phase of the disease all lend support to attempt APC or PC infusion in severe AP.

## Conclusion

In summary, the current study demonstrated significant PC pathway pathology in severe AP. The PC pathway defects were more frequent in patients developing MOF.

## Key messages

• 	Severe AP patients suffer from significant PC pathway pathology, which is associated with the development of organ failure.

• 	Monocyte HLA-DR% correlated with PC and APC levels, indicating interaction between inflammation and the PC pathway in severe AP.

• 	Modulating the PC pathway might improve the outcome of severe AP patients.

## Abbreviations

AP = acute pancreatitis; APC = activated protein C; HLA = human leukocyte antigen; IL = interleukin; mAb = monoclonal antibody; MOF = multiple organ failure; PC = protein C; PT = prothrombin time.

## Competing interests

The authors declare that they have no competing interests.

## Authors' contributions

OL and LK executed the study and drafted the manuscript. LK, JAF, JHG, PP, HR, and JP participated in the original design and coordination of the study, and in writing the original protocol. JAF and JHG carried out the PC and APC assays. LK, PM, HR, and JP analysed the data. PM, EK, PP, HR, and JP assisted in drafting the manuscript. RH assisted in the original design and drafting of the final manuscript. All authors read and approved the final manuscript.

## Figures and Tables

**Table 1:** Characteristics of patients with acute pancreatitis either with (cases, *n *= 13) or without (controls, *n *= 18) organ failure

Variable	Cases	Controls
Age	47 (31–83)	44 (24–75)
Gender (male/female)	12/1	11/7
Aetiology		
Alcohol	11	15
Biliary	2	3
Length of hospitalization (days)	35 (4–130)	13 (2–90)
Length of the stay in the intensive care unit (days)	26 (2–129)	6 (2–20)
Mechanical ventilation	12	
Length of mechanical ventilation (days)	10 (4–129)	
Haemodialysis	5	
Need for inotropes	10	
Died of acute pancreatitis	3	

Data presented as median (range) or number of patients.

**Table 2:** Coagulation parameters and the proportion of positively fluorescing monocytes (HLA-DR%) in patients with organ failure (cases) and control patients during the stay in the intensive care unit

Variable	Cases	Controls	*P *value
Lowest activated protein C level (%)	85 (76–102)	97 (73–136)	0.009
Lowest protein C level (%)	52 (8–70)	70 (18–113)	0.03
Lowest activated protein C/protein C ratio	1.1 (0.87–13.2)	1.3 (1.0–3.8)	0.02
Highest activated protein C/protein C ratio	1.8 (1.5–13.2)	1.6 (1.2–4.1)	0.03
D-dimer (mg/l)	5.5 (1.1–40.1)	4.7 (1.4–47.3)	0.22
Prothrombin time (%)	77 (17–147)	77 (13–138)	0.10
Platelet count (× 10^9^/l)	185 (48–738)	208 (40–530)	0.38
HLA-DR%	46 (8–82)	44 (11–84)	0.80

Data presented as median (range).
